# Progress Toward Eliminating Mother to Child Transmission of HIV in Kenya: Review of Treatment Guidelines Uptake and Pediatric Transmission Between 2013 and 2016—A Follow Up

**DOI:** 10.1007/s10995-018-2612-0

**Published:** 2018-07-25

**Authors:** Ruby Angeline Pricilla, Melinda Brown, Catherine Wexler, May Maloba, Brad J. Gautney, Sarah Finocchario-Kessler

**Affiliations:** 1 Department of Community Medicine, Low Cost Effective Care Unit, Christian Medical College, Schell Eye Hospital Campus, Vellore, Tamil Nadu India; 2 Department of Family Medicine, University of Kansas Medical Center, Kansas City, KS USA; 3 Global Health Innovations, Kansas City, USA

**Keywords:** Preventing mother to child transmission (PMTCT), WHO guidelines, Option B+, Gestational week of initiation, Pediatric HIV transmission

## Abstract

*Background* Prevention of mother to child transmission of HIV (PMTCT) services are critical to achieve national and global targets of 90% antiretroviral therapy (ART) coverage in PMTCT, and mother to child transmission rates less than 5%. In 2012, Kenya adopted WHO’s recommended ART regimen for PMTCT “Option B+”. *Aims* This study assesses progress made in adopting these new guidelines and associated outcomes. *Methods* We analysed programmatic data of 2604 mother–infant pairs enrolled in the HIV Infant Tracking System (HITSystem) at four government hospitals in Kenya between January, 2013 and December, 2016. We then compared PMTCT trends between 2010 and 2012 and 2013–2016 for the same four government hospitals. *Results* A total of 2,371 (91.1%) received some ART regimen, however; only 911 (56.2%) mothers received ART regimens compliant with WHO Option B+. From 2013 to 2016, the percent of mothers on WHO Option B + doubled from 42 to 84% (p < 0.001), the mean week of ART initiation decreased from 19.0 to 9.7 weeks (p < 0.001), the percent of pregnant women who were already on ART at the time of PMTCT enrolment increased from 5.8 to 31.7% (p < 0.001), and the paediatric transmission rate decreased from 5.9 to 2.5% (p = 0.002). *Conclusion* Comparing data at these four Kenyan hospitals indicates significant progress has been made from 2010 to 2016. To continue these positive gains, concerted focus will be needed to target and improve the integration of new guidelines into clinical practice at the facility level, adherence to treatment and retention in care.

## Significance

Kenya has made significant investments to expand the coverage and quality of PMTCT services, evidenced by the decline in infant HIV transmission from 16% in 2012 to 8.3% in 2015. Kenya supported rapid adoption of the WHO PMTCT treatment guidelines, which have evolved with research and scientific advances. PMTCT data from four government hospitals from 2013 to 2016 were analysed to assess implementation of WHO and national guidelines, identify challenges, and measure outcomes for HIV-exposed infants. We then compared these data to previously published data from the same four hospitals from 2010 to 2012 to measure longer term progress (2010–2016) in Kenya.

## Introduction

In 2015, the UNAIDS reported 2.1 million new HIV infections globally for a total of 36.7 million people living with HIV (UNAIDS 2016). Approximately 1.1 million of these new cases were from Eastern and Southern Africa (UNAIDS 2016) where new HIV infections attributable to perinatal transmission have decreased from 18% in 2010 to 6% in 2015 (AVERT 2017). Kenya has made significant investments to expand the coverage and quality of prevention of mother to child transmission (PMTCT) services (PEPFAR 2016), increasing the proportion of HIV+ pregnant women receiving antiretroviral therapy (ART) from 60% in 2013 to 75% in 2015 (Kenya Ministry of Health 2016a). While this investment in PMTCT services has contributed to decreasing rates of mother to child transmission (from 16% in 2012 to 8.3% in 2015) (Kenya Ministry of Health 2016a), Kenya has yet to achieve the global target of reducing perinatal transmission to 5% (UNAIDS 2011). The Kenyan National AIDS and STI Control Programme has supported rapid adoption of the WHO PMTCT treatment guidelines, which have evolved with research and scientific advances over time (Kenya Ministry of Health 2016a).

In 2010, WHO guidelines recommended that HIV positive pregnant women with CD4 counts ≤ 350/mm^3^ or clinical stage 3, 4 were treated with triple ART regimen. For those with CD4 counts > 350 and clinical stage 1, 2 there were 2 option for prophylaxis: option A included Zidovudine (AZT) started as early as 14 weeks of gestation in the antepartum, single dose Nevirapine (NVP) and initiation of AZT/3TC (lamivudine) in the intrapartum and continuation of AZT/3TC for 7 days in the postpartum periods. Option B included triple ART regimen started as early as 14 weeks of gestation and continued until delivery or 1 week after cessation of breastfeeding (World Health Organization 2010). In 2012, the WHO adopted “Option B+”, a single universal regimen to treat HIV infected pregnant women to prevent mother to child transmission (MTCT) of HIV (World Health Organization 2012). Option B+ includes a triple ART regimen prescribed as soon as pregnant women are diagnosed (at any gestational age), with continued treatment for life in settings which have capacity to implement and monitor triple therapy. With this change, treatment was no longer dependent on the CD4 counts or clinical staging, thus minimizing barriers and delays to treatment initiation in low resource settings and reducing the risk of drug resistance caused by stopping and restarting ART with each pregnancy (World Health Organization 2012). This significant change to the guidelines was intended to optimize maternal health and prevention of HIV transmission during current and future pregnancies. In the same year (2012), Kenya adopted the WHO guidelines and began implementing the shift to Option B+ regimens for HIV infected pregnant women to prevent MTCT (Kenya Ministry of Health 2012).

**Fig. 1:**
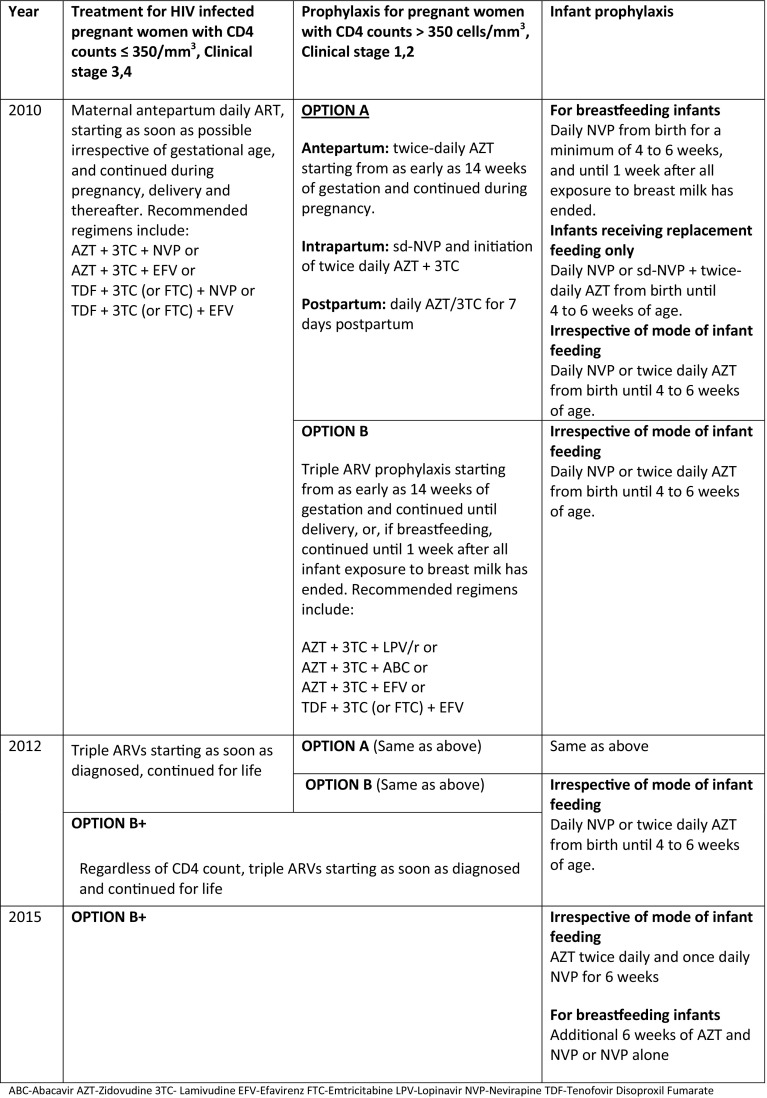
WHO PMTCT guidelines 2010, 2012, 2015

Similarly, Kenya adopted the additional WHO modifications to the PMTCT guidelines in 2016 (Kenya Ministry of Health 2016b), which included 12 weeks of infant prophylaxis with Nevirapine (NVP) as recommended by the WHO (World Health Organization 2016). Figure 1 demonstrates the changes in WHO PMTCT treatment guidelines over time (World Health Organization 2010, 2012, 2016).

Dissemination and adoption of revised clinical guidelines into practice is a multistep process that requires updates to first be integrated into national guidelines and then implemented at the facility level. By 2016, only 19 out of 33 sub-Saharan Africa countries had integrated the WHO 2012 guidelines into their national guidelines and only 3 had integrated the 2015 guidelines, taking an average of 19 and 8 months to do so, respectively (Gupta and Granich 2016). Furthermore, once integrated into guidelines, implementation at the facility level is often suboptimal. An evaluation of PMTCT services between 2010 and 2012 at four government hospitals in three counties (Nairobi, Nandi and Kericho) in Kenya revealed that the average gestational age at ART initiation decreased significantly from 26.6 weeks in 2010 to 14.5 weeks in 2012, but less than 20% of HIV-positive pregnant women received the Option B+ regimen, and HIV infection rates among infants born to HIV-positive women averaged 5.9% (Finocchario-Kessler et al. 2016). The current study analyses PMTCT data from the same four hospitals during the subsequent 4 year period (2013–2016) to assess progress made in implementing WHO and national guidelines, challenges with implementation, and outcomes for HIV-exposed infants. We then compare PMTCT trends between these two periods of time at the same four government hospitals.

## Methods

Programmatic data were extracted from four government hospitals implementing the HIV Infant Tracking System (HITSystem) as part of the standard of EID (early infant diagnosis) care since 2012. All mother–infant pairs who presented for EID care and enrolled in the HITSystem between 2013 and 2016 at one of the study hospitals were eligible for enrolment in the study.

As described previously (Finocchario-Kessler et al. 2014), the HITSystem is an online intervention implemented to support and track early infant diagnosis (EID), linkage to treatment, and retention of HIV-exposed infants through 18 months of age. At the time of infant’s enrolment in EID, mother’s demographic information (age, education level) and antenatal history including gestational week of ART initiation and ART regimen (pre/intra/postpartum) were retrospectively entered into the HITSystem. The HITSystem then prospectively tracked infant outcomes (prophylaxis for opportunistic infections (OI) and HIV, age at first HIV test, polymerase chain reaction (PCR) test result, infant feeding method, and all other relevant EID services). All data points analysed were collected in the online HITSystem for the study duration. The HITSystem uses numeric IDs for patient information, thus all patient details were de-identified prior to analysis. The study protocol was approved by the Institutional Review Board at the Kenya Medical Research Institute (SERU#1890).

### Outcomes and Measures

The primary outcomes measured were maternal ART regimen and gestational week of initiation. Women already on ART prior to pregnancy were reported as initiating at zero gestational weeks. The infant HIV transmission rate was calculated from the first postnatal HIV PCR result and compared across year of EID enrolment to determine the trends in pediatric HIV transmission. The timing of infant HIV testing, type and duration of infant postnatal ART prophylaxis, OI prophylaxis, and infant feeding method at the time of the first HIV test were also analysed.

### Statistical Analysis

Data from the four hospitals were exported to Excel files generated by the HITSystem. The data were cleaned and analysed using SPSS version 23. Frequencies and percentages were calculated for categorical variables and mean, median, standard deviation (SD) were calculated for continuous variables. We compared the mean week of ART initiation between years using *t* tests. The proportion of mothers who received Option B+ during pregnancy and perinatal HIV transmission rates were compared between years using Chi square test. The likelihood of perinatal HIV transmission during 2013–2016 by type of regimen and gestational timing of ART initiation were analysed using Chi square test. A p-value of < 0.05 was considered as the level of significance.

## Results

A total of 2604 mother–infant pairs were enrolled in the HIT System at the four hospitals from January 2013 to December 2016. Demographic data for the mothers were limited (42% with maternal age and 47% with education level recorded). For those with recorded data, mean maternal age at the time of EID enrolment was 29 years (SD: 5.68; range 15–49). For level of education, 27% completed primary, 26% completed high school, and less than 2% attended college/university education. Exclusive breastfeeding (92.8%) was the most common method of feeding at the time of infant testing.

### Adherence to PMTCT Guidelines, 2013–2016

A majority of the mothers (2371, 9.1%) received some ART regimen during pregnancy (Table 1). A total of 1620 (68.3%) of mother–infant pairs had data on gestational week of ART initiation. Of these, 552 (34.1%) received ART late (≥ 14 weeks gestational age). There were 911 (56.2%) mothers who received ART regimens compliant with WHO Option B+ during pregnancy. *Majority of the* infants received postpartum ART prophylaxis (95.9%) and co-trimoxazole (98.8%), of whom more than half (54.1%) received NVP for 12 weeks in the postpartum period.

**Table 1:** A comparison of adherence and trends in PMTCT guidelines between 2010 and 2012 and 2013–2016

Variables	2010–2012	2013–2016	X^2^	p value
Mother–infant pairs	1365	2604		
ART regimen				
Mothers on any ART	1134 (83.1%)	2371 (91.1%)	55.18	< 0.001
Late ART initiation	752 (66.3%)	552 (34.1%)	278.12	< 0.001
On time initiation of ART^a^				
WHO A	160 (14.1%)	157 (9.7%)	124.54	< 0.001
WHO B+	222 (19.6%)	911 (56.2%)		
Infant postpartum prophylaxis				
Received ART prophylaxis	1267 (92.8%)	2497 (95.9%)	17.24	< 0.001
NVP × 12 weeks		1350 (54.1%)		
NVP × 6 weeks	533 (42.1%)	636 (25.5%)	108.14	< 0.001
NVP × 6 months	694 (54.8%)	429 (17.2%)	567.47	< 0.001
Other regimen	40 (3.2%)	82 (3.2%)	0.04	0.84
Infant OI prohylaxis				
Received co-trimoxazole	1359 (99.6%)	2574 (98.8%)	5.06	0.02
Infant HIV testing				
Infants tested for HIV	1347 (98.7%)	2553 (98.0%)	2.15	0.14
Median infant age (in weeks) at 1st PCR	6.86	6.0		
	SD 14.41	SD 12.44		
Infants testing HIV +	79 (5.9%)	109 (4.3%)	4.89	0.03
Infants with indeterminate HIV test result	6 (0.4%)	36 (1.4%)	7.70	< 0.01
Infant feeding method	n = 1330	n = 2487		
Breast feeding only	1122 (84.4%)	2308 (92.8%)	49.56	< 0.001
Replacement feeding only	94 (7.1%)	66 (2.7%)	42.04	< 0.001
Mixed feeding	114 (8.6%)	113 (4.5%)	19.55	< 0.001

^a^Weeks of gestation recorded for 1620 mothers only

### Trends in PMTCT Guideline Implementation, 2013–2016

**Fig. 2:**
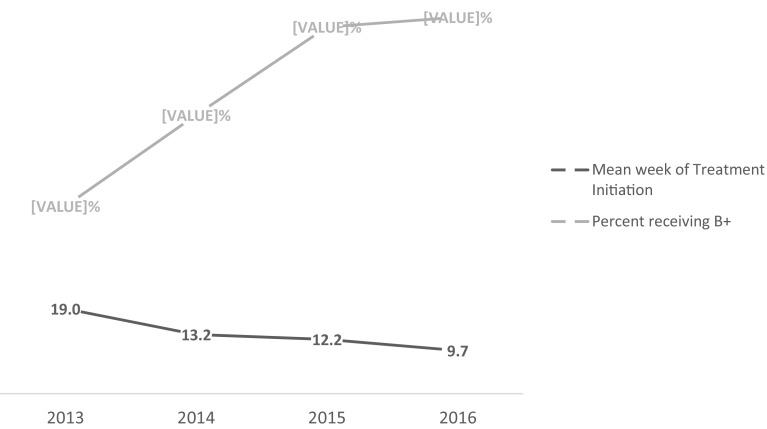
Trends in PMTCT implementation guideline in Kenya (2013–2016)

Figure 2 illustrates gestational age at ART initiation and type of regimen by year. The proportion of mothers who received WHO Option B+ during pregnancy doubled from 42% in 2013 to 84% in 2016 (x^2^ = 256.8, p < 0.001). The mean gestational age at initiation of ART for the 4 year period of 2013–2016 was 13.3 gestational weeks (SD 9.90, median = 14), decreasing from 19.0 weeks in 2013 to 9.7 weeks in 2016 (t = 14.69, p < 0.001). The proportion of mothers who initiated ART at a late gestational age (≥ 14 weeks) decreased from 34.0% in 2013 to 19.3% in 2016 (x^2^ = 35.13, p < 0.01). Across all 4 years, 19.5% of mothers who were already on ART at the time of enrolment into PMTCT. This increased significantly from 5.8% in 2013 to 31.7% in 2016 (x^2^ = 145.10, p < 0.001).

Table 2 compiles the *t* test results for the mean week of ART initiation, x^2^ and p-values for the Option B+ between the years. There were significant decrease in the mean week of ART initiation from 2013 to 2014 and 2015 to 2016, but not from 2014 to 2015. The proportion of mothers who received the recommended Option B+ during pregnancy increased significantly from 2013 to 2014 and 2014 to 2015, but not from 2015 to 2016.

**Table 2:** Trends in PMTCT implementation guideline in Kenya year wise 2013–2016

	Change in mean	*t* test results	p value
Week of ART initiation			
2013–2014	5.8	8.38	< 0.001
2014–2015	1.0	1.72	0.08
2015–2016	2.5	3.95	< 0.01

	% change	X^2^	p value
Option B+			
2013–2014	20.4	54.47	< 0.001
2014–2015	19.7	61.29	< 0.001
2015–2016	2.2	1.17	0.28
Infant HIV positive test result			
2013–2014	0.8	0.47	0.49
2014–2015	1.9	2.76	0.97
2015–2016	0.7	0.60	0.44
Infant HIV indeterminate result			
2013–2014	1.1	3.87	0.05
2014–2015	0.4	0.41	0.52
2015–2016	1.2	2.81	0.09

### Trends in Infant HIV Testing and Transmission, 2013–2016

**Fig. 3:**
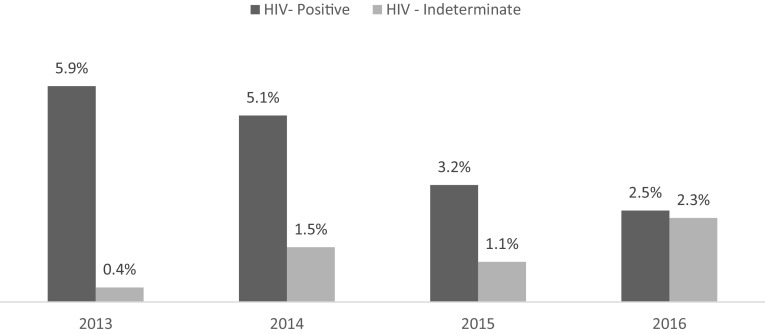
HIV+ DNA PCR test results among HIV—exposed infants enrolled in EID by year, % (2013–2016)

The mean age of HIV PCR testing for HIV exposed infants in EID was 8.57 weeks (SD = 12.44). Test results were available for the majority (98%) of infants, with 109 (4.3%) infants testing positive and 36 (1.4%) infants with an indeterminate test result (Table 1). From 2013 to 2016, there was a decrease in the transmission rate from 5.9% in 2013 to 2.5% in 2016 (x^2^ = 9.87, p = 0.002). There was a gradual increase in HIV indeterminate results from 0.4% in 2013 to 2.3% in 2016 (x^2^ = 8.56, p = 0.003) (Fig. 3). Of the 36 infants with indeterminate PCR test results, 27 (75%) received confirmatory re-tests, yielding 5 HIV-positive test results. The likelihood of infant HIV transmission (Table 3) was significantly higher among mothers who did not receive any ART during pregnancy (x^2^ = 87.25, p < 0.001), who started on Option A compared to Option B+ (x^2^ = 6.27, p = 0.01), and who initiated ART after 14 weeks gestation (x^2^ = 3.99, p = 0.04).

**Table 3:** Likelihood of HIV transmission compared by type of PMTCT & timing of intervention—2013–2016

	HIV positive test results	Chi square	p value
Type of ART regimen			
No ART (n = 233)	37 (15.9%)		
Mother on any ART (n = 2371)	72 (3.0%)	87.25	< 0.001
Option A (n = 421)	21 (5.0%)		
Option B+ (n = 1883)	50 (2.7%)	6.27	0.01
Time of initiation			
Late initiation > 14 weeks of gestation (n = 552)	22 (4.0%)	3.99	0.04
On time initiation 0–14 weeks of gestation (n = 1068)	24 (2.2%)		

### Comparing 2010–2012 and 2013–2016 Findings (Table 1)

There was a significantly higher proportion of mothers who received any ART regimen during pregnancy (91.1 vs 83.1%, x^2^ = 55.18, p < 0.001) and the desired Option B+ regimen (56.2 vs 19.6%, x^2^ = 124.54, p < 0.001) in 2013–2016 as compared to 2010–2012. The proportion of mothers who initiated ART for PMTCT at a late gestational age (≥ 14 weeks) decreased significantly from 66.3% (2010–2012) to 34.1% (2013–2016), x^2^ = 278.12, p < 0.001, but nearly a third (31.7%) were missing data on the gestational week of ART initiation in 2013–2016. The proportion of infants receiving postpartum ART prophylaxis increased significantly from 92.8% (2010–2012) to 95.9% (2013–1016), x^2^ = 17.24, p < 0.001. In 2013–2016, more than half of infants received ART prophylaxis according to the new guidelines: NVP for 12 weeks, which was not prescribed in the previous years. Test results were available for nearly all (98.0%) of the infants, which was similar to the 2010–2012 data (98.7%). The median infant age at first PCR test decreased from 6.86 weeks in 2010–2012 to 6.0 weeks in 2013–2016. The proportion of infants testing positive for HIV decreased significantly from 5.9% in 2010–2012 to 4.3% in 2013–2016, x^2^ = 4.89, p = 0.03, but the proportion of infants who had an indeterminate HIV test result increased significantly from 0.4% (2010–2012) to 1.4% (2013–2016), x^2^ = 7.70, p < 0.01. There was a significant increase in the proportion of breastfed infants (92.8 vs 84.4%), x^2^ = 49.56, p < 0.001, while the proportion of infants on replacement feeding and mixed feeding was lower in 2013–2016 compared to 2010–2012 (2.7 vs 7.1%) and (4.5 vs 8.6%), respectively.

## Discussion

The UNAIDS 90–90–90 Targets (90% of people living with HIV who know their status, 90% who receive ART, and 90% who have suppressed viral loads) (World Health Organization 2016) are essential for the goal of ending the AIDS epidemic by 2030. By 2015, Kenya hoped to achieve 90% PMTCT coverage and reduce mother to child transmission of HIV to less than 5% (UNICEF 2012). The findings of this paper clearly demonstrate a promising trend in these four government hospitals in Kenya. National level data suggests that the overall use of ART in pregnancy is 75% (Kenya Ministry of Health 2016a) in Kenya and the average PMTCT coverage of the three counties to which these hospitals belonged was high; 89% in Kericho, 97% in Nairobi and 99% in Nandi (NASCOP 2016). In our study, the proportion of mothers who received any ART regimen during pregnancy increased significantly from 83.1% in 2010–2012 to 91.1% in 2013–2016. Furthermore, there was a promising increase in the proportion of mother who received Option B+ during pregnancy from 42 to 84.3% between 2013 and 2016. While these data indicate that some regions of Kenya has achieved the target of 90% ART coverage during PMTCT, ensuring more widespread and consistent use of the optimal Option B+ regimen is needed to further reduce rates of MTCT (NASCOP 2016).

Although Kenya has adopted the latest WHO guidelines and continues to increase PMTCT coverage, achieving *early* initiation of Option B+ in pregnancy remains an important focus. While there was a significant increase in the proportion of mothers who were already on ART at the time of PMTCT enrolment between 2013 and 2016, and a corresponding decrease in the mean week of ART initiation from 19.0 to 9.7 weeks; about one-third of women still initiated ART late (≥ 14 weeks).

These data demonstrated that women who were on any ART, and those initiated on Option B+ on time, were significantly less likely to transmit HIV. Importantly, infant HIV transmission across the four hospitals decreased from 5.9% in 2010–2012 to 4.3% in 2013–2016; representing the achievement of national targets to reduce MTCT to < 5% by 2019 (PEPFAR 2016). In 2016, pediatric transmission rates averaged from these hospitals reached a low of 2.5%.These findings are comparable to the 2.8% transmission rate reported by the PMTCT-HEI survey conducted in 62 facilities across Kenya in 2016 (Nganga et al. 2016), yet are comparatively lower than the 2016 county level estimates for Kericho (9%), Nandi (5%) and Nairobi (3.7%) (NASCOP 2016); which are not limited to clinic-based populations. The increase in indeterminate results from 2013 to 2016 is concerning, as this presents opportunities for missed diagnoses if infants cannot be traced and a new sample cannot be collected. There is some evidence suggesting that maternal and/or infant exposure to ART for PMTCT can suppress infant VL and lead to indeterminate results (Mazanderani et al. 2014). As access to PMTCT expands under evolving guidelines, it is important to ensure that diagnostic tools and algorithms also evolve to best diagnose infants exposed to highly effective ART through PMTCT.

Furthermore, the latest WHO guidelines (2015) recommends 6 weeks of AZT twice daily and NVP for 6 weeks and an additional 6 weeks of AZT and NVP or NVP alone for breastfed HIV-exposed infants (12 weeks total) (World Health Organization 2016). Kenya was quick to implement this latest recommendation, which was reflected in 54.1% of the HIV-exposed infants receiving NVP for a total of 12 weeks at the study hospitals during 2015–2016. In our study 25% of HIV exposed infants received NVP for 6 weeks which was expectedly lower than the 53.3% reported by the 2016 PMTCT-HEI survey (Nganga et al. 2016). Overall, these data reflect a significant improvement in Kenya’s PMTCT services. Delays in early initiation of ART during pregnancy (Gupta and Granich 2016), limited monitoring of medication adherence, and insufficient tracking and support for retention throughout pregnancy and the postpartum period are persisting challenges for PMTCT services.

### Strengths and Limitations

These data clearly reflect the positive trends in adopting the latest PMTCT guidelines by these four hospitals in Kenya. Generalizability may be limited as this study only looked at mother–infant pairs enrolled in the HIT System, which is an intervention that targets women who have already presented for EID care at hospitals, thus does not include women who are not accessing EID services who are arguably at increased risk for suboptimal HIV outcomes. Counselling mothers during ANC/PMTCT care about the importance of infant testing and what to expect has been associated with increased EID uptake and timely infant testing (Goggin et al. 2016). Furthermore, innovative strategies for EID demand creation and community-based infant HIV testing are needed (Macharia et al. 2018). Since maternal characteristics were retrospectively entered at the time of infant enrolment into EID, data regarding mother’s age, level of education and other individual level variables that could have influenced utilization of PMTCT services. The antenatal week of ART initiation was also not documented well, which could have influenced the observed trends in timing of ART initiation. Finally, measuring ART adherence and postpartum retention in care is critical to providing high quality PMTCT to eliminate mother to child HIV transmission. While the current HITSystem targets EID outcomes, an adapted version has been developed to support PMTC outcomes (appointment attendance, medication adherence, hospital delivery and early linkage to EID), which will improve documentation of maternal characteristics, improve enrolment in EID services by automatically linking PMTCT and EID services, and help target the remaining gaps in Kenya’s PMTCT efforts.

## Conclusion

This study provides evidence that Kenyan hospitals are moving in the right direction with regard to PMTCT coverage, provision of the recommended Option B+ regimen, and earlier timing of ART initiation. Compared to earlier analyses, a higher proportion of infants received HIV prophylaxis, as well as HIV testing. Most importantly, the proportion of infants infected with HIV has decreased. Kenya must protect and expand these gains in their PMTCT services, while strengthening strategies to improve monitoring and support for medication adherence and retention in care. Moving forward, these pillars of optimal PMTCT will need increased focus to actualize elimination of pediatric HIV.

## References

[CR1] AVERT. (2017). PMTCT of HIV (2017). https://www.avert.org/professionals/hiv-programming/prevention/prevention-mother-child. Accessed 10 January 2018.

[CR2] Finocchario-Kessler S, Clark KF, Khamadi S, Gautney BJ, Okoth V, Goggin K (2016). Progress toward eliminating mother to child transmission of HIV in Kenya: Review of treatment guideline uptake and pediatric transmission at four government hospitals between 2010 and 2012. AIDS and Behavior.

[CR3] Finocchario-Kessler S, Gautney BJ, Khamadi S, Okoth V, Goggin K, Spinler JK (2014). If you text them, they will come: Using the HIV infant tracking system to improve early infant diagnosis quality and retention in Kenya. AIDS.

[CR4] Goggin K, Wexler C, Nazir N, Staggs VS, Gautney B, Okoth V (2016). Predictors of infant age at enrollment in early infant diagnosis services in Kenya. AIDS and Behavior.

[CR5] Gupta S, Granich R (2016). When will sub-Saharan Africa adopt HIV treatment for all?. Southern African Journal of HIV Medicine.

[CR6] Kenya Ministry of Health. (2012). *Guidelines for PMTCT of HIVAIDS in Kenya*. https://www.faces-kenya.org/wp content/uploads/2012/11/Guidelines-for-PMTCT-of-HIVAIDS-in-Kenya-1_2012.pdf. Accessed February 21, 2018.

[CR7] Kenya Ministry of Health. (2016a). *Kenya AIDS Response Progress Report*. http://nacc.or.ke/wp-content/uploads/2016/11/Kenya-AIDS-Progress-Report_web.pdf. Accessed February 21, 2018.

[CR8] Kenya Ministry of Health. (2016b). *Guidelines on use of antiretroviral drugs for treating and preventing HIV infection in Kenya*. https://www.faces-kenya.org/wp-content/uploads/2016/07/Guidelines-on-Use-of-Antiretroviral-Drugs-for-Treating-and-Preventing-HIV.pdf. Accessed February 21, 2018.

[CR9] Macharia, L., Brown, M., Wexler, C., & Finocchario-Kessler, S. (2018). Community feedback on point of care HIV testing for Infants and children in Kenya, presented at the Global Health Conference Midwest, 2018. Omaha, NE, USA.

[CR10] Mazanderani AF, du Plessis NM, Thomas WN, Venter E, Avenant T (2014). Loss of detectability and indeterminate results: Challenges facing HIV infant diagnosis in South Africa’s expanding ART programme. SAMJ: South African Medical Journal.

[CR11] NASCOP. (2016). *Kenya HIV county profiles*. http://nacc.or.ke/wp-content/uploads/2016/12/Kenya-HIV-County-Profiles-2016.pdf. Accessed February 21, 2018.

[CR12] Nganga, L., McGrath, C. J., & Langat, A. (2016). Mother-to-child transmission of HIV in Kenya: A multi-year national evaluation. PMTCT-HEI survey in 2016. Retrieved from http://www.croiconference.org/sessions/mother-child-transmission-hiv-kenya-multiyear-national-evaluation.

[CR13] PEPFAR. (2016). *Kenya country operational plan (2016)*. https://www.pepfar.gov/documents/organization/257644.pdf. Accessed February 21, 2018.

[CR14] UNAIDS. (2011). *Global plan elimination HIV in children*. http://www.unaids.org/sites/default/files/media_asset/20110609_JC2137_Global-Plan-Elimination-HIV-Children_en_1.pdf. Accessed February 21, 2018.

[CR15] UNAIDS. (2016). *Global AIDS update*. http://www.unaids.org/en/resources/documents/2016/Global-AIDS-update-2016. Accessed January 10, 2018.

[CR16] UNICEF. (2012). *Countdown to zero*. https://www.unicef.org/french/aids/files/hiv_pmtctfactsheetKenya.pdf. Accessed February 21, 2018.

[CR17] World Health Organization. (2010). *Antiretroviral drugs for treating pregnant women and preventing HIV infection in infants*. http://apps.who.int/iris/bitstream/10665/75236/1/9789241599818_eng.pdf. Accessed February 21, 2018. 26180894

[CR18] World Health Organization. (2012). *Use of antriretroviral drugs for treating pregnant women and preventing HIV infections in infants*. http://www.who.int/iris/handle/10665/70892. Accessed February 21, 2018.

[CR19] World Health Organization. (2016). *Consolidated guidelines on the use of antiretrovial drugs for treating and preventing HIV infection*. 27466667

